# The Genetic Architecture Underlying the Evolution of a Rare Piscivorous Life History Form in Brown Trout after Secondary Contact and Strong Introgression

**DOI:** 10.3390/genes9060280

**Published:** 2018-05-31

**Authors:** Arne Jacobs, Martin R. Hughes, Paige C. Robinson, Colin E. Adams, Kathryn R. Elmer

**Affiliations:** 1 Institute of Biodiversity, Animal Health and Comparative Medicine, College of Medical, Veterinary and Life Sciences, University of Glasgow, Glasgow G12 8QQ, Scotland, UK; a.jacobs.1@research.gla.ac.uk (A.J.); martin.hughes.biology@outlook.com (M.R.H.); paigecr@hotmail.co.uk (P.C.R.); colin.adams@glasgow.ac.uk (C.E.A.); 2 Scottish Centre for Ecology and the Natural Environment, University of Glasgow, Rowardennan, Loch Lomond, Glasgow G63 0AW, Scotland, UK

**Keywords:** adaptation, genomic divergence, ferox trout

## Abstract

Identifying the genetic basis underlying phenotypic divergence and reproductive isolation is a longstanding problem in evolutionary biology. Genetic signals of adaptation and reproductive isolation are often confounded by a wide range of factors, such as variation in demographic history or genomic features. Brown trout (*Salmo trutta*) in the Loch Maree catchment, Scotland, exhibit reproductively isolated divergent life history morphs, including a rare piscivorous (ferox) life history form displaying larger body size, greater longevity and delayed maturation compared to sympatric benthivorous brown trout. Using a dataset of 16,066 SNPs, we analyzed the evolutionary history and genetic architecture underlying this divergence. We found that ferox trout and benthivorous brown trout most likely evolved after recent secondary contact of two distinct glacial lineages, and identified 33 genomic outlier windows across the genome, of which several have most likely formed through selection. We further identified twelve candidate genes and biological pathways related to growth, development and immune response potentially underpinning the observed phenotypic differences. The identification of clear genomic signals divergent between life history phenotypes and potentially linked to reproductive isolation, through size assortative mating, as well as the identification of the underlying demographic history, highlights the power of genomic studies of young species pairs for understanding the factors shaping genetic differentiation.

## 1. Introduction

Understanding the genomic architecture underlying the evolution and maintenance of reproductive isolation and phenotypic divergence in the face of gene flow is a major question in evolutionary biology. Over the last decade, numerous studies have documented genomic patterns of differentiation among recently evolved species, life history types or ecotypes [1,2,3,4,5,6,7,8,9,10,11]. Traditionally, heterogeneous patterns of genetic differentiation across the genome and associated peaks of elevated differentiation were interpreted to have mainly arisen due to ecological specialization and reproductive isolation [12,13,14,15,16]. These peaks were hypothesized to contain barrier loci resistant to gene flow, whereas the remainder of the genome was homogenized by gene flow [12,13,14,15,16]. However, such regions of elevated relative genetic differentiation (Fst) may also arise and be structured by a variety of processes, such as sorting of ancient polymorphisms, variation in demographic and evolutionary history, recent selection, and reduced diversity or background selection in low recombination regions [14,15,16,17,18,19,20,21,22,23]. Variation in a single factor or combinations of these factors has been associated with variable genetic differentiation across the genome [14,15,20,24,25], diluting the signal of differentiation caused by loci associated with reproductive isolation. The geographic mode of speciation, such as secondary contact versus primary divergence with gene flow, also has a strong impact on the expected patterns of differentiation; under secondary contact genomic islands of high differentiation can form as a result of selection in allopatry or by the erosion of large-scale differentiated regions after secondary contact [22,26,27]. Furthermore, reduced recombination, e.g., in centromeric regions, or increased background selection in regions with high gene density have been shown to decrease local genetic diversity and therefore cause increased relative genetic differentiation (‘Fst’) [16,18]. Measures of among-group genetic diversity, such as Fst and Dxy (an absolute measure of genetic divergence), are differentially affected by within-group genetic diversity (e.g., π or gene diversity). Furthermore, differential sorting of ancient polymorphisms into species will still lead to increased absolute genetic divergence, causing the formation of a heterogeneous landscape of genetic differentiation and divergence [21,28,29]. However, in allopatry or under secondary contact with long allopatric phases, increased relative and absolute differentiation (Fst and Dxy) are expected [18]. Thus, in order to understand how genomic islands form and their role in the formation and maintenance of reproductive isolation and adaptive phenotypic divergence, it is crucial to first understand the evolutionary history of a system and account for different driving factors by using a range of different measures.

Many salmonid species display distinct trophic and life history forms that differ in ecology, morphology and behavior [30,31,32,33,34] and show varying levels of genetic differentiation [31,32,35]. In some cases, it has been shown that these distinct ecotypes or life history forms have most likely evolved due to secondary contact of postglacial lineages, such as lake whitefish (*Coregonus clupeaformis*) and European whitefish (*Coregonus lavaretus*) [36,37]. Similar to many other polymorphic species, these ecotypes often display heterogeneous genetic landscapes of divergence across the genome [7,11], but to date these patterns have only been assessed in detail in a few species. Brown trout (*Salmo trutta*) is a widespread salmonid species in the northern hemisphere that displays a number of different life history strategies. These include anadromy, in which individuals migrate to sea after a period as juveniles in freshwater streams (such as sea trout), and adfluvial potamodromy, in which, following a period in streams, individuals migrate into lakes until they become sexually mature and migrate back to the natal stream for spawning. The most common foraging strategy among this latter group is to feed on littoral zoobenthos [38] yet in some large, deep and oligotrophic lakes adfluvial potamodrous brown trout may exhibit a relatively rare piscivorous life history [39,40,41,42]. Although piscivorous life histories have also evolved in other salmonid species, they are typically found in very low abundances within populations compared to other sympatric life history forms [30,34,43,44,45,46]. This rare life history, colloquially referred to as a “ferox” life history (hereafter ferox trout), manifests predominantly in the occupation of the pelagic lacustrine habitat and piscivorous trophic niche [42,47,48,49]. As adults, ferox trout have increased body size [50,51], delayed maturation [52] and increased longevity [50,51] compared to the common benthivorous lacustrine brown trout (hereafter benthivorous brown trout). Juvenile ferox trout also showed increased dominance and food acquisition over benthivorous brown trout in laboratory experiments [49], suggesting a faster growth as juveniles, which is important for quickly reaching the large body size and consequent gape-size necessary for a piscivorous life style [49,53,54]. In some lakes in Scotland and Ireland, ferox trout have been shown to be reproductively isolated and genetically distinct from sympatric benthivorous brown trout, but the geographic range of sites where this has been tested is very limited [40,55,56,57,58,59]. Based on the genetic distinctiveness in some lakes, ferox trout have been classified as a distinct species (*Salmo ferox* Jardine, 1835), as nomenclature recognized by the by the International Union for the Conservation of Nature (IUCN) [60]. However, due to the limited knowledge regarding the genetic basis of the ferox life history, they are classified as data deficient [60]. Relatively high mortality of hybrids between benthivorous brown trout and piscivorous ferox trout from the Maree catchment [49] suggests that these life history forms are not only reproductively isolated by extrinsic barriers but also that intrinsic post-zygotic barriers, such as genetic incompatibilities, may be operating at least in this catchment [12,32,61,62,63,64]. These post-zygotic barriers have been observed in other sympatric salmonid ecotypes [62,65].

Here, we investigate the genetic architecture and evolutionary history underlying the divergence between two life history forms in brown trout (*Salmo trutta*), the benthivorous brown trout and piscivorous ferox trout, from the Maree catchment in northern Scotland. Using genome-wide SNPs from ddRADseq and mitochondrial haplotypes, we show for the first time that these genetically distinct life history forms have evolved under secondary contact and remain partially reproductively isolated despite extensive gene flow and introgression. We find strong genetic differentiation in several genomic regions across the genome, some of which have formed through selection or reduced introgression. We identify several loci and regions under selection, within and outside of genomic outlier windows, and further identify several associated candidate genes and functions potentially involved in the eco-morphological and life history divergence between benthivorous brown trout and ferox trout.

## 2. Materials and Methods

### 2.1. Sampling

**Figure 1:**
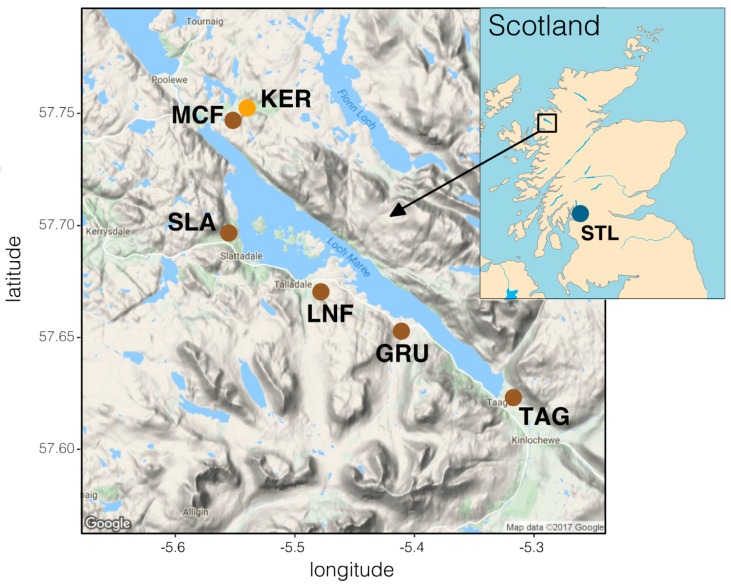
Map of sampling sites in Scotland and the Maree catchment. Benthivorous brown trout (brown dots) were sampled from Taggan (TAG), River Grudie (GRU), Loch na Fideil (LNF), River Slattadale (SLA) and the MacFarlane burn (named for this study, MCF). Ferox trout from the Maree catchment (orange dot) were sampled from the Kernsary river (KER). Sampling sites of sea trout from Loch Lomond (STL) are marked with a blue dot. An overview of sample numbers is given in Table 1.

Ferox trout and benthivorous brown trout were collected from six tributaries of Loch Maree. Fish were caught by non-lethal sampling using fyke nets and electrofishing in October and November 2013 as fish migrated from Loch Maree into spawning tributaries [49]. For all fish collected an adipose fin clip was taken and stored in 100% ethanol for later analysis. All ferox trout from the Maree catchment in this study were collected from the Kernsary river, as no other spawning sites for ferox trout are known from this catchment. Trout were primarily classified as ferox trout or benthivorous brown trout based on fork length (ferox: 40–80 cm, benthivorous brown trout: 20–35 cm) [39,40,49]. This classification was then confirmed using stable isotope analyses of eggs taken from mature individuals (see [49] for details). Additionally, three anadromous brown trout (sea trout), collected by electro-fishing from Loch Lomond (identified using the criteria of [66]) were obtained as additional samples for the mitochondrial DNA analysis. A summary of sampling sites and sample sizes is available in Figure 1 and Table 1. One of the sampling sites, an un-named tributary of the Kernsary river was allocated a name for the purposes of this paper (McFarlane or MCF). Although benthivorous brown trout and ferox trout are sympatric during the feeding season, they spawn in different locations (in parapatry or allopatry).

### 2.2. Library Preparation and Sequencing

Genomic DNA was extracted from adipose fin clips using a column extraction kit (NucleoSpin Tissue, Macherey Nagel, Düren, Germany) following the manufacturer’s recommended protocols. DNA quality was assessed by visualization after gel electrophoresis on a 2% Agarose gel and quantified using a Qubit 2.0 Fluorometer with the dsDNA BR Assay (Life Technologies, Carlsbad, CA, USA). Each sample was normalized to a total amount of 1 µg of DNA with a minimum concentration of 25 ng/µL. DNA was used to construct a single ddRAD-library containing 44 individuals, following a previously described protocol in [67] with a modified set of barcoded P1 and P2 adapters for paired-end sequencing on Illumina platforms. In brief, each sample was digested using two restriction enzymes, a rare-cutting enzyme (PstI-HF, 20 units) and a frequent-cutting enzyme (MspI, 20 units) (New England Biolabs, Ipswich, MA, USA). Samples were individually barcoded using unique combinations of barcoded P1 and P2 adapters. After multiplexing of the barcoded samples, a size selection step was performed using the Pippin Prep (Sage Science, Beverly, MA, USA) and fragments between 145–295 bp were selected. The amplified library was sequenced on half a lane (200 million reads) of an Illumina NextSeq 500 (75 bp paired-end reads) at *Glasgow Polyomics* (University of Glasgow).

### 2.3. ddRAD Genotyping and Bioinformatics

Raw reads were demultiplexed and trimmed to 60 bp using the “process_radtags” script in *Stacks* v 1.46 [68]. Data are available in the NCBI Sequence Read Archive [SRP149094]. The paired-end reads for each individual were mapped against the Atlantic salmon reference genome ([69]; ICSASG_v2) using *Bowtie2* v.2.2.6 [70] with the *very-sensitive* setting with all other settings at default and reads with mapping qualities (MAPQ) below 20 were subsequently removed using *samtools* v 1.3.1 [71]. Because no brown trout reference genome is available [72] and our markers were not compatible with the available brown trout linkage map [73], the Atlantic salmon reference genome was used. This genome is informative because Atlantic salmon is in the same genus and shows large-scale synteny with brown trout [74,75,76]. We used the “*ref_map.pl*” pipeline in *Stacks* v 1.46 to call SNPs in each individual and filtered the dataset with a minimum stack depth of three (m = 3). The “*rxstacks*” correction module in *Stacks* was used to remove loci that were confounded in more than 25% of individuals or showing an excess of haplotypes, based on the frequencies of haplotypes in the entire population. The haplotype pruning step only keeps the two most frequent haplotypes for each individual. Furthermore, genotypes were called using a 5% significance level cut-off and an upper bound of 0.05 for the error rate, and genotype calls with a summed log-likelihood for each locus and individual of less than −10 were removed. Genotype calls were exported for subsequent analyses using the populations module in *Stacks* with the following filtering thresholds: a minor-allele frequency (MAF) of 0.05, a maximum observed heterozygosity of 0.6 to remove potential paralogous loci, a stack depth of three, and a SNP had to be present in at least 66% of individuals within a population and in 4 out of 6 populations (sampling sites). We did not implement a specific Hardy-Weinberg filtering step due to strong admixture among populations. To assess the effect of relative low stack depth on population structuring, we also extracted a SNP dataset using the same filtering criteria but with a stack depth of six (m = 6). The MAF filtering step was not implemented for the demographic inferences in order to retain informative low frequency polymorphisms and only SNPs with less than 10% missing data were used for building the site frequency spectrum. Only the first SNP per locus was used for all downstream analyses to reduce the impact of linkage on the subsequent analyses. We furthermore exported haplotype information for the subset of filtered SNPs to use the information of all SNPs within a RAD locus to analyze genetic diversity across the genome.

### 2.4. Population Genomic Analyses

*Admixture* v. 1.3 [77] was used with a tenfold cross-validation to perform individual clustering and assess admixture proportions between different benthivorous brown trout spawning sites, Kernsary ferox trout, and Loch Lomond sea trout. The *Admixture* analysis was also performed for the dataset with a higher stack depth. Furthermore, we performed a principal component analysis (PCA) using the R package *adegenet* [78] to analyze and visualize the major axes of genetic variation. Missing data for the PCA were inferred using the default settings by replacing them with mean allele frequency values. To quantitatively assess the level of population structuring and differentiation, we performed an AMOVA in *Genodive* [79] with populations (sampling sites) nested within life history types (benthivorous brown trout vs. ferox trout). Furthermore, we calculated genome-wide summary statistics, namely number of private sites, average nucleotide diversity and observed heterozygosity for each population in the Maree catchment using *Stacks* based on all variant and invariant sites. We further assessed the genetic relationship among individuals, populations and ecomorphs by creating a neighbor-joining network using *SplitsTree* v. 4.14.5 [80].

### 2.5. Correlation between Ancestry Coefficient and Morphology

To assess the correlation between genetic ancestry and morphology, we calculated the spearman’s rank correlation coefficient between the genetic ancestry coefficient inferred with *Admixture* and fork length, a phenotypic trait that strongly distinguished the different life history types [39,49]. Fork length was not age-adjusted, but all fish used for the analyses were sexually mature.

### 2.6. Inferring the Divergence History of Benthivorous Brown Trout and Ferox Trout Evolution

To assess the evolutionary history of ferox trout and benthivorous brown trout from the Maree catchment, we used the joint site frequency spectrum and coalescence simulations implemented in *fastsimcoal v.2.5.2* [81]. *fastsimcoal2* is a composite-likelihood-based approach and performs coalescence simulations based on a predefined model and subsequently uses a conditional maximization (ECM) algorithm for optimizing each parameter to maximize the data-based estimated likelihood. We calculated the joint site frequency spectrum (JSFS) used for the demographic modelling using a differently filtered SNP dataset without a minor allele frequency threshold and a maximum of 10% missing data. The SFS was calculated using *∂a∂i* v.1.6.3 [82]. We compared the divergence history between benthivorous brown trout individuals combined across spawning sites, excluding individuals with more than 25% ferox ancestry, and individuals classified as ferox trout from the Kernsary river. The final dataset for this analysis contained 20 benthivorous brown trout and 8 ferox trout. We used the folded joint site frequency spectrum (JSFS) and projected it down to 12 and 6 samples for benthivorous brown trout and ferox trout, respectively, to reduce the impact of missing genotypes. To be able to determine absolute divergence times, we corrected the number of monomorphic sites in the site frequency spectrum as described in [83], as only one SNP per locus was used for this analysis, biasing the ratio of polymorphic to monomorphic sites in the JSFS and therefore the inferred parameter estimates. The number of monomorphic sites theoretically equals the number of loci times the length of each locus (55 bp; 60 bp reads minus the 5 bp restriction site) minus the number of segregating sites. Therefore, we calculated the actual ratio of polymorphic to monomorphic sites using all segregating sites and multiplied the number of segregating sites (N = 11,088 SNPs) used for inferring the JSFS by this inferred ratio to obtain the adjusted number of monomorphic sites for the JSFS [83]. We further used a mutation rate of 1 × 10^−8^ [37], since no accurate mutation rate for brown trout is available.

We compared six different divergence models: Strict Isolation (*SI*), Isolation-with-migration (*IM*), Ancient Migration (*AM*), Secondary Contact (*SC*), Isolation-with-migration with change in migration rate (*IMchange*) and Secondary Contact with admixture (*SCadm*). The first five models (*SI, IM, AM, SC, IMchange*) are commonly used models for inferring divergence histories [84,85]. The *SI* model describes the divergence between two lineages without any gene flow, while the *IM* model describes a divergence history under constant gene flow (a single symmetric gene flow rate was estimated). The *AM* and *SC* model describe ancient gene flow that stopped at some point in time or recent gene flow that started following the split of two lineages without gene flow, respectively. The *IMchange* model is a variation of the *IM* model, in which the rate of symmetric gene flow changes at some point in time. The *SCadm* model is a variation of the *SC* model, that also estimates the proportion of admixture at the time of secondary contact [83,85]. As it is not possible to accurately infer the directionality of gene flow from the folded JSFS, we only modelled a single migration rate between both groups. We initially conducted 25 independent runs for each model to assess convergence and detect the most likely model. Each run was conducted with 40 rounds of parameter estimation with 100,000 coalescence simulations. We used the Akaike information criterion (AIC), adjusted for the number of parameters in a model, to select the most likely model [81]. To obtain the best parameter estimates and confidence intervals (CI) for each parameter, we performed an additional 55 runs for the best model and used the parameters from the top ten runs with the lowest estimated likelihood for non-parametric bootstrap resampling [86]. We estimated parameter means and 95 percentiles confidence intervals from bootstrap distributions based on 100,000 resampling steps using the *resample* R package [87].

Furthermore, to test the effect of population structuring across the pooled benthivorous brown trout populations and unequal sample sizes for benthivorous brown trout and ferox trout on the demographic model selection, we performed the same demographic analysis only for individuals from the LNF sampling site. We chose this site as it had a similar number of samples as the ferox trout. We projected the JSFS down to six individuals for both groups and corrected the number of monomorphic sites in the same way as for the full dataset. We ran each model 25 times and selected the best model as explained above.

### 2.7. Genome-Scans and Genomic Outlier Regions

We analyzed Fst and nucleotide diversity (π) using SNPs, and gene diversity and haplotype diversity using haplotypes, within and between benthivorous brown trout and ferox trout across the genome. We only used non-admixed benthivorous brown trout (N = 20; with benthivorous brown trout genetic ancestry above 0.75 inferred using *Admixture* with K = 2) and ferox trout from the Kernsary river. We also excluded two benthivorous brown trout individuals from the McFarlane burn that indicated 100% “Ferox-ancestry” from this analysis, as we could not be absolutely sure that these were benthivorous brown trout. Pairwise Fst and nucleotide diversity were inferred using *vcftools* [88] using the filtered SNP dataset. We z-transformed the SNP-based Fst-values and used a threshold of ZFst > 4 for detecting putative outlier loci, which is equivalent to the 99.17% quantile of the Fst distribution. The z-transformed Fst value (ZFst) gives the number of standard deviations from the mean (Fst value for each SNP) and allowed the standardized identification of outlier loci. Furthermore, haplotype-based gene diversity and haplotype diversity for each RAD locus were inferred using *Stacks*. In contrast to nucleotide diversity, the haplotype-based diversity measures take the full diversity within the RAD locus into account, and more than two haplotypes can exist for a locus. Gene diversity is the probability that two randomly chosen haplotypes for a given locus are different, and haplotype diversity, is scaled to the substitution distance between two randomly chosen haplotypes [68,89,90]. Values were smoothed and plotted along the genome using the loess smoothing method (span = 0.1) in the *geom_smooth* function of *ggplot2*.

We also performed a sliding window analysis with a window size of 500 kb (average of 4 SNPs per window) and a step size of 125 kb to identify larger genomic regions that are significantly differentiated between ferox and benthivorous brown trout. We z-transformed the window-based mean Fst and defined outlier windows as those with a mean ZFst above 4 and containing at least two differentiated variants. Even though this approach might suffer a loss of power due to the relatively low density of SNPs along the genome, we used it to identify larger regions that were consistently differentiated. Furthermore, we compared the nucleotide diversity, gene diversity and haplotype diversity within and outside of those outlier windows using a Wilcoxon rank sum test. To assess the influence of functional density (density of annotated regions), a proxy for the density of targets of selection, on patterns of genetic diversity across the genome, we compared the density of annotated characters in the Atlantic salmon genome (Annotation Release 100) within and outside of genomic outlier windows using a Wilcoxon rank sum test. We calculated the functional density as the number of annotated characters within 500 kb sliding windows and a step size of 125 kb. We grouped all windows that overlapped with genomic outlier windows. We furthermore assessed the correlation between functional density and Fst using a Spearman’s rank correlation implemented in the *cor.test* R function.

To assess the consistency of genomic outlier windows across different population pairs and the impact of unequal sample sizes on Fst estimation, we performed window-based genome scans by sampling site. We performed separate genome scans for Kernsary ferox trout vs. benthivorous brown trout from GRU, LNF or MCF. We only used non-admixed individuals for LNF (N = 11) and GRU (N = 7), and only the four individuals without complete ferox ancestry from MCF. We plotted the loess smoothed ZFst values for each window across the genome and qualitatively compared their overlap with the identified genomic outlier windows. Furthermore, we performed SNP-based genome scans and calculated Spearman’s correlations for Fst values (based on individual SNPs) for all pairwise comparisons to assess the correlation of genetic differentiation across comparisons.

Additionally, we used *pcadapt* v.3.0.4 [91], a principal component-based eigen-GWAS approach [92,93], to detect loci that differentiate along the first two eigenvectors and are putatively under selection. Compared to the window-based genome scan, this approach is based on individual SNPs and is therefore less affected by the relatively low SNP density. Furthermore, *pcadapt* was shown to have a low false discovery rate that is comparable to other state-of-the art approaches such as *hapFLK* [94]. It is powerful in detecting outliers under population divergence scenarios, even with admixture, and can detect outlier loci without defining discrete populations [91]. We analyzed the overlap between the genome scan outlier loci and loci putatively under selection (*pcadapt* outlier) to determine which outlier loci are also genetically differentiated between ferox trout and benthivorous brown trout and are located within genomic outlier windows.

### 2.8. Outlier Annotation and Over-Representation Analysis

To identify potential candidate genes involved in the divergence between ferox trout and benthivorous brown trout, we first identified all genes in the Atlantic salmon (*Salmo salar*) genome annotation [69], ICSASG_v2; Annotation Release 100) overlapping or closest (<50 kb) to loci putatively under selection (associated with principal component 1). Second, we identified all genes within genomic outlier windows. Using all identified candidate genes within genomic outlier windows, we performed an over-representation analysis in *WEBGESTALT* [95] for geneontology (biological processes) terms and KEGG pathways. This analysis was performed based on zebrafish orthologues and using the zebrafish (*Danio rerio*) genome as background to identify statistically enriched pathways that might play an important role in the phenotypic and life history divergence between ferox trout and benthivorous brown trout.

Furthermore, to indirectly infer potential functional genomic regions, we used a publicly available database of QTL for six salmonid species and their location on the Atlantic salmon genome [96] to identify QTL, derived from Atlantic salmon studies, that overlap with genomic outlier windows and loci under selection. We used Atlantic salmon derived QTL, as no QTL information are available for brown trout. Atlantic salmon is the most closely related species with QTL information on phenotypic traits that differ between benthivorous brown trout and ferox trout, such as body length and weight.

### 2.9. Mitochondrial DNA: Sequencing and Analysis of the ND1 Gene

The ND1 gene was amplified for 39 individuals using the primers *B1NDF* and *B1NDR* [97], using the same PCR conditions as described in [97]. The PCR product was sent to *DNA Sequencing and Services* (MRC I PPU) for cleaning and Sanger sequencing in both directions. Forward and reverse contigs were assembled and trimmed to a common length using the *CLC Bio Genomics Workbench* v.6.5 (CLC bio, Aarhus, Denmark). Data are available in GenBank [MH400439-MH400477]. Sequences were aligned using *Muscle* [98] implemented in *MEGA* v.7 [99].

To analyze population structure based on the mitochondrial ND1 gene, a TCS haplotype network was constructed using *POPART* [100,101].

## 3. Results

### 3.1. Genome-Wide Dataset and Summary Statistics

A total of 214,995 loci were obtained for all individuals based on an average of 2.5 ± 0.98 (mean ± SD) million mapped reads per individual (mean coverage = 11.5 ± 4.1), containing a total of 40,715 SNPs. After stringent filtering (see Methods), a global dataset of 16,066 SNPs was retained for 35 individuals (Table 1) with an average of 12.5 ± 14.7% missing data per individual (mean ± SD). Seven individuals were excluded due to low sequencing coverage.

### 3.2. Population Structure and Admixture

**Figure 2:**
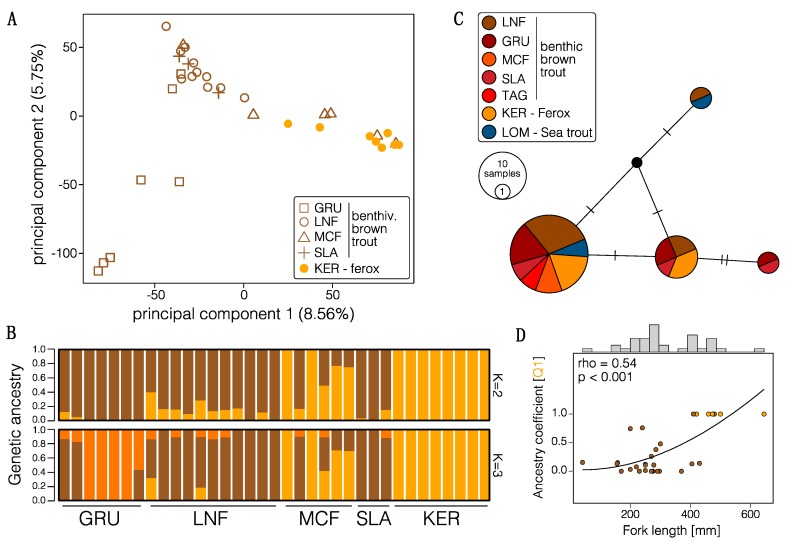
Population structure of brown trout. (**A**) Principal component plot showing the first and second axis of variation. (**B**) Proportion of genetic ancestry for two (K = 2) and three (K = 3) genetic clusters. Population codes are explained in Table 1. (**C**) A TCS-haplotype network based on the mitochondrial ND1 gene. (**D**) Correlation between genetic ancestry and fork length in brown trout from the Maree catchment. The grey area around the loess-smoothed line shows the standard error. Ferox trout (orange) had an average fork length of 483 ± 72 mm and benthivorous brown trout (brown) had an average fork length of 267 ± 88 mm. Values for the analysis were not age-corrected, as information on the age of individuals was not available.

In order to examine the population structure and degree of reproductive isolation among benthivorous brown trout and ferox trout, we used several different approaches. The genetic clustering analysis revealed the presence of two predominant clusters (K = 2; cv = 0.634) that mainly differentiate the ferox trout from the Kernsary river from all other individuals (Figure 2B). However, we find two individuals from the adjacent McFarlane burn with complete ”ferox genetic ancestry” and relatively high proportions in the other individuals from this spawning site (Figure 2B), indicating strong gene flow and incomplete reproductive isolation among ferox trout and benthivorous brown trout in parapatry. We only find relatively low admixture proportions in allopatric brown trout populations. Benthivorous brown trout from Grudie share a distinct cluster at K = 3 (cv = 0.676; Figure 2B). Using the SNP dataset filtered with a stack depth of six (13,854 SNPs) we recovered the same genetic ancestry proportions, suggesting that our results are not strongly biased towards benthivorous brown trout alleles, despite the low ferox sample size and stack depth (data not shown). Overall, the proportion of genetic ancestry is positively correlated with fork length in individuals from the Maree catchment (Spearman’s rank correlation = 0.54, *p* < 0.001; Figure 2D).

The principal component analysis revealed a similar result (Figure 2A), with principal component (PC) 1 mainly separating the Kernsary ferox trout from all other populations (PC1 explains 8.56% of the overall variation) and PC2 (5.75%) separating Grudie individuals from all other benthivorous brown trout individuals (Figure 2A). The clustering of two Kernsary ferox individuals with benthivorous brown trout from the McFarlane burn can be explained by slightly higher amount of missing data in those two individuals that were replaced with mean allele frequencies, however that does not affect the clustering of all other individuals that have lower amounts of missing data. We observed a generally high level of admixture, particularly in adjacent populations (Figure 2). Similar clustering was observed in the neighbor-joining network (Figure S1). The nested AMOVA supported these results, showing a significant structuring by location but no significant structuring by life history type), with an overall low but significant level of genetic differentiation among populations (Fst = 0.074, *p* < 0.001; Table 2).

The mitochondrial ND1 gene revealed the presence of four haplotypes but no significant structuring by population or life history (Figure 2C), suggesting strong admixture among populations or a common founding population. None of the haplotypes were population or life history type specific, and most populations contained more than one single haplotype.

### 3.3. Divergence History of Benthivorous Brown Trout and Ferox Trout

**Figure 3:**
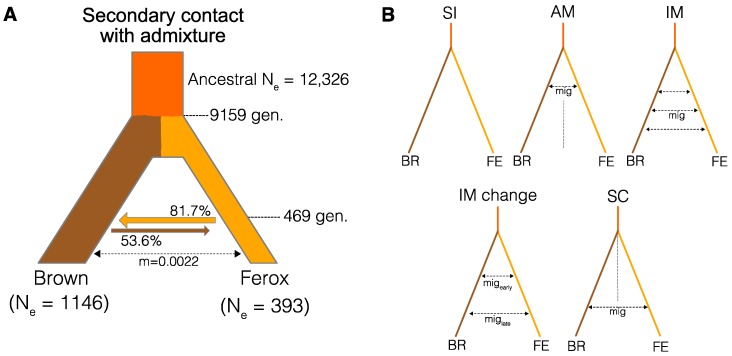
Evolutionary history of divergence between ferox trout and benthivorous brown trout. (**A**) Illustration of the most likely model of divergence between ferox trout and benthivorous brown trout. Point estimates for all estimated parameters are given. Confidence intervals are given in Table 4. (**B**) Illustrations of the remaining tested models between benthivorous brown trout (BR) and ferox trout (FE).

To assess the divergence history of ferox trout and benthivorous brown trout from the Maree catchment, we implemented a coalescence modelling approach and compared the fit of six different divergence models (Figure 3) to the empirical data using *fastsimcoal2* and the joint site frequency spectrum (JSFS; Figure S2). Incorporating contemporary gene flow into the model (*SC, IM, IMchange, SCadm*) always improved the fit of the model compared to the strict isolation (*SI*) and ancient migration (AM) model (Table 3). The secondary contact with admixture model (Figure 3A, *SCadm*), under which ferox trout and benthivorous brown trout split followed by a phase without gene flow before secondary contact and admixture, was the most likely model (∆AIC to next best model “*IMchange*” = 73). In general, the *SCadm* model generally fit well to the data based on the relatively small difference between the maximum estimated likelihood (−47,407.865) and the maximum observed likelihood for the given JSFS (−47,395.854).

Furthermore, performing the demographic modelling on a trimmed dataset without pooling benthivorous brown trout populations (only used the LNF population) also selected the *SCadm* model as the most likely divergence scenario (Table S1; ∆AIC to next best model “*IMchange*” = 8.7). Although the difference between the models was smaller, which could be explained by the reduced amount of information in the dataset, it shows that population structuring in benthivorous brown trout did not have a significant effect on the demographic model selection. Therefore, we focused our interpretation and parameter estimation on the model including all brown trout populations.

The estimated divergence time between benthivorous brown trout and ferox trout under the “*SCadm*” model for the combined dataset was 9159 (8271–10292) generations with a recent timing of secondary contact 469 (445–498) generations ago (Figure 3A; Table 4). Using an average generation time of 4 years, as ferox trout mature on average later (5+ years) than benthivorous brown trout (2-4+ years) [52], this translates into a divergence time of around 36,363 (33,084–41,168) years and a recent timing of secondary contact around 1876 (1780–1992) years ago. The inferred admixture proportions revealed strong introgression between ferox and benthivorous brown trout on secondary contact with up to 81.7% (80.6–82.7%) admixture. The estimated admixture proportions showed that admixture from ferox into benthivorous brown trout was on average significantly stronger (81.7%) than vice versa (53.6%). Inferred effective population sizes (*Ne*) of benthivorous brown trout (1146 individuals) were approximately 3 times higher compared to ferox trout (393), which is expected based on the rarity of ferox trout [42,46] or could also be due to the difference in generation time between ferox trout and benthivorous brown trout [50,51,52]. Detailed estimates and confidence intervals for all estimated parameters are given in Table 4 and Figure 3. In general, the estimated parameters, such as divergence times, have to be interpreted with caution due to the use of a generic mutation rate and the strong difference in the generation times between life history forms.

### 3.4. Outlier Analysis and Genome-Scans

**Figure 4:**
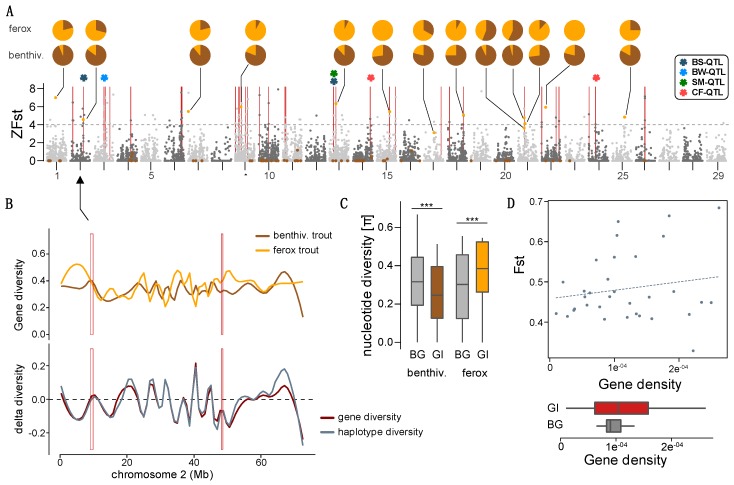
Genomic patterns of differentiation across the genome. (**A**) Window-based genetic differentiation (ZFst) across the Atlantic salmon genome. Note that the Atlantic salmon genome only has 29 chromosomes compared to 40 chromosomes in the brown trout genome [73]. We set negative ZFst values to zero for plotting purposes. Genomic outlier windows are highlighted with red bars. QTL derived from Atlantic salmon [96] that overlap with genomic outlier windows are highlighted by stars. BS: body shape, BW: body weight, SM: Timing of sexual maturation, CF: Condition factor. *Pcadapt* outlier loci associated with PC1 are highlighted in orange, and those associated with PC2 in brown. The pie charts are showing the allele frequencies of *pcadapt* outlier loci associated with PC1, differentiating ferox trout and benthivorous brown trout. (**B**) Patterns of haplotype-based gene diversity and delta diversity across chromosome 2. Negative delta diversity shows reduced diversity in benthivorous brown trout and vice versa. The second genomic outlier window on this chromosome shows reduced genetic diversity in benthivorous brown trout compared to ferox trout, indicative of a selective sweep in benthivorous brown trout. See Figure S3-S5 for all chromosomes. (**C**) Patterns of genetic diversity (π) in genomic outlier windows versus the genomic background in benthivorous brown trout and ferox trout. (**D**) Gene density is not correlated to the average degree of genetic differentiation of genomic outlier windows (Spearman’s rank correlation, r = 0.09, *p* = 0.618). Furthermore, gene density does not differ between genomic outlier windows and the genomic background (Wilcoxon test, *p* = 0.8385).

Using a *pcadapt* analysis, we detected a total of 96 loci that differentiated along the first two principal components and are candidates for being under selection (Figure 4A). Thirteen SNPs differentiated individuals along PC1 and 83 loci along PC2. As the PCA inferred with *pcadapt* (not shown) was equivalent to the PCA inferred with *adegenet* we assumed that loci differentiating the Kernsary ferox trout from benthivorous brown trout will mainly be associated with PC1 (Figure 2A). The genome scan showed that 11 out of the 13 *pcadapt* outlier SNPs were also significantly differentiated between ferox trout and benthivorous brown trout (ZFst > 4; Figure 4A). Most of these SNPs were widely distributed across the genome (across 11 chromosomes), except three outlier loci, which were located within a 2.5 Mb region on chromosome 21 (Figure 4A). PC1 outlier loci had a mean Fst of 0.66 ± 0.128 compared to a mean genome-wide Fst of 0.071 ± 0.117. Outlier loci associated with PC2 on the other hand were not differentiated between ferox trout and benthivorous brown trout (mean Fst = 0.041 ± 0.043), suggesting that loci associated with PC1 are potentially underlying the phenotypic diversification between ferox trout and benthivorous brown trout in the Maree catchment.

Furthermore, Fst-values showed a right-tailed distribution with several loci across the genome showing moderate to high differentiation (Figure S6). None of the differentiated SNPs were fixed between ferox and benthivorous brown trout. Using a sliding-window approach, we detected 33 genomic outlier windows across 16 of the 29 Atlantic salmon chromosomes, of which four contained outlier loci identified using *pcadapt*.

To assess the effect of unequal sample sizes, geographic distance and clustering of structured populations on the detection of genomic outlier windows, we estimated sliding window-based Fst values between Kernsary ferox trout and benthivorous brown trout from LNF, MCF and GRU separately. We found that genomic outlier regions detected using combined populations were also differentiated in most pairwise comparisons, with some degree of variation (Figure S7). In general, patterns of pairwise Fst (calculated based on individual SNPs) were significantly correlated across all comparisons (Spearman’s correlations, FE-GRU vs. FE-LNF: r = 0.37; FE-GRU vs. FE-MCF: r = 0.26; FE-LNF vs. FE-MCF: r = 0.30; *p* < 0.001), but with more distant populations showing higher degrees of genetic differentiation (Figure S7). However, compared to the combined genome scan, we found several fixed SNPs between benthivorous brown trout and ferox trout in the GRU vs. KER (N_fixed_ = 1) and the MCF vs. KER (N_fixed_ = 5) comparisons, although they were not shared across comparisons. Overall, this suggests that geographic distance and sample size played a small but non-significant role in the detection of genomic outlier windows.

In order to disentangle if these outlier windows arose as an artefact of reduced diversity or potentially due to selection, we examined patterns of inter- and intrapopulation genetic diversity across the genome (π, gene diversity, haplotype diversity, and Fst) and compared them between genomic outlier windows and the remainder of the genome (genomic background). Patterns of nucleotide diversity showed that most genomic outlier windows were on average located within regions of reduced diversity in benthivorous brown trout (Wilcoxon rank sum test: *p* < 0.001) and increased diversity in ferox trout (Wilcoxon rank sum test: *p* < 0.001) compared to the genomic background (Figure 4C), suggesting potential selective sweeps in benthivorous brown trout. However, there is extensive variation with several outlier windows being located in regions with reduced diversity in both ecotypes (Figure 4B, Figure S2–S4), suggesting that these genomic windows were formed due to locally reduced genetic diversity. Since nucleotide diversity was only calculated based on SNPs and can therefore give biased estimates, we used haplotype-based genetic diversity as a complementary approach. Using these haplotype-based estimates of genetic diversity, we found similar patterns of genetic diversity across the genome, and between genomic outlier windows and background, compared to nucleotide diversity. Although gene diversity in genomic outlier windows was reduced compared to the genomic background in brown trout, but not in ferox trout, this comparison was not significant (Wilcoxon test: *p* = 0.0575). Furthermore, patterns of haplotype-based genetic diversity across the genome confirmed that some genomic outlier windows were located in regions with reduced diversity in one of the forms (differential delta diversity; Figure 4B; Figures S3–S5). We found that 9 genomic outlier windows were located within regions of reduced diversity in brown trout compared to ferox trout (negative delta diversity) and 7 genomic outlier windows in regions of reduced diversity in ferox trout (positive delta diversity). This suggests that increased genetic differentiation in those 16 genomic windows was potentially caused by selection and not only overall decreased genetic diversity in low-recombination regions. The remaining 17 genomic outlier windows are most likely artefacts of locally reduced diversity, for example due to increased background selection.

To further test for the potential influence of background selection on genetic differentiation, we tested if the density of functional regions (gene density) is increased within genomic outlier windows compared to the genomic background, as (ii) gene dense regions have a higher probability of experiencing background or positive selection, (ii) gene density is potentially positively correlated with recombination rate [14] and (iii) gene density has been shown to be positively correlated with density of quantitative trait loci in salmonids [96]. However, the mean gene density within genomic outlier windows did not significantly differ compared to the genomic background (Figure 4D; Wilcoxon test, *p* = 0.8385), suggesting that background selection is not higher in genomic outlier windows compared the genomic background and does not explain increased differentiation in those regions. Gene density differed between genomic outlier windows, but this was not explained by differences in genetic differentiation (Spearman’s rank correlation, r = 0.09, *p* = 0.618; Figure 4D) suggesting that variation in gene density was not driving patterns of differentiation in this species.

### 3.5. Annotation of Pcadapt Outlier Loci and Genomic Outlier Windows

Outlier loci detected using *pcadapt* were located within genes or close to genes involved in a range of molecular functions and biological processes, such as immune response (*pik3R5*; *tlr2; pip4k2c; igsf3*), global gene expression regulation (*rrp1B*), lipid localization (*gulp1*) and muscular development (*pdzrn3*) (Tables S2–S4). Although genes within genomic outlier windows were not significantly enriched for specific KEGG pathways (Table S3) or biological processes (GO terms; Table S2), we found that genes were involved in a range of biological processes related to growth and development, such as the transforming growth factor beta (TGF) pathway (GO:0007179; GO:0071559; GO:0071560), or (transmembrane) transport (GO:0055085; GO:0034220; GO:0006810; GO:0006811), negative regulation of gene expression (GO:0010629), RNA catabolism (GO:0006401) and localization (GO:0051234). Many of these processes and pathways associated with genomic outlier windows or genes under selection, such as the TGF pathway (growth and development), lipid localization and immune response could potentially explain the phenotypic and behavioral differences between ferox trout and benthivorous brown trout, such as increased growth and active swimming behavior. However, these results are only suggestive due to the low SNP density and further analyses using high-density SNP datasets and ideally more population replicates are necessary to get a robust idea of the functional genomic basis underlying the ferox life history.

Furthermore, we found that five genomic outlier windows overlapped with QTL for body shape, body weight, condition factor and timing of sexual maturation, which were derived from Atlantic salmon [96], suggesting that these genomic regions are potentially involved in the divergence of those phenotypic traits.

## 4. Discussion

Our results reveal a heterogeneous genomic landscape differentiating the rare piscivorous ferox and benthivorous brown trout life history forms that have evolved recently under secondary contact of two distinct postglacial lineages. The genomic landscape of differentiation is characterized by moderately to highly differentiated genomic outlier windows that are scattered across the genome, including several loci showing signs of selection. By comparing patterns of diversity, differentiation and density of functional elements in those genomic outlier windows to the genomic background, we found evidence that at least some of these genomic regions were formed under selection or reduced introgression and potentially contribute to adaptive divergence and reproductive isolation among life history forms. We further identified several genes that are differentially selected and pathways that are potentially involved in phenotypic divergence and reproductive isolation, such as genes involved to development, growth, and gene expression regulation.

Different life history types and eco-morphological specialists, such as piscivorous and benthivorous Arctic charr (*Salvelinus alpinus*) [35,43], beach versus stream spawning sockeye salmon (*Oncorhynchus nerka*) [11,102], dwarf versus normal lake whitefish (*Coregonus clupeaformis*) [32,103] or benthic and limnetic European whitefish [33,104], have evolved in a range of salmonid species, with varying levels of reproductive isolation. Although in some cases intrinsic reproductive barriers have evolved [62], as it is likely the case between ferox trout and benthivorous brown trout in the Maree catchment [49], differences in body size have been shown to strongly affect assortative mating in brown trout and other fish species [105,106], therefore potentially linking reproductive isolation and trophic specialization. This might be particularly important as piscivorous individuals are larger than conspecifics with differing ecological specializations, since body size and related traits such as gape width are important factors determining the foraging success of piscivorous individuals [49,53,54]. The correlation of body size with the proportion of genetic ancestry (Figure 2D) suggests that body size and correlated traits might be at least partially genetically inherited. However, the association is mostly driven by the larger size of ferox trout rather than strong variation within benthivorous brown trout making it difficult to clearly determine the impact of genetic ancestry proportion on body size. Although a similar pattern could also evolve neutrally, we expect differences in body size to be adaptive for piscivorous foraging [49,53,54]. Therefore, it is important to study reproductive isolation and eco-morphological and life history adaptation combined, as these can be strongly linked.

Understanding the demographic history underlying the evolution of different trophic specialists and life histories is essential for untangling factors shaping the genomic landscape of differentiation [26]. Similar to other polymorphic salmonid populations [37], the ferox life history in the Maree catchment has most likely evolved after the secondary contact of two glacial refugia lineages that split before the last glacial maximum around 33,084–41,168 years ago which then came into contact recently, around 1780–1992 years ago (Figure 3A). We have no good explanation for the very recent timing of secondary contact, but the timings of divergence and secondary contact have to be interpreted with caution due to the use of average generation times and a generic mutation rate. Nonetheless, the inferred divergence time corresponds to a divergence between the two lineages before the last glacial maximum in the British Isles around 27,000–21,000 years ago [107]. Our results also suggest that secondary contact only occurred recently, around 8000–10,000 years after the Pleistocene glaciers retracted from northern Scotland [107]. In general, secondary contact has also been suggested for ferox trout in Loch Awe and Lough Melvin based on phylogeographic comparisons [59]. The inferred evolutionary history suggests a built-up of genome-wide genetic differentiation in allopatry before secondary contact, and could explain the evolution of intrinsic barriers to hybridization, manifesting in relatively high hybrid mortality [49]. However, strong admixture at the time of secondary contact (41.8–82.7% admixture), is a likely explanation for the relatively low contemporary background divergence as well as the absence of population structure based on the mitochondrial ND1 gene (Figure 2C and Figure 4A), as introgression of mitochondrial DNA can be relatively strong in salmonids [108]. The asymmetry in admixture between ferox trout and benthivorous brown trout upon secondary contact could potentially be explained by size-assortative mating and the often observed higher reproductive success of larger (ferox) individuals [106,109], although it is difficult to infer the driving factors. The evolutionary history of secondary contact with recent but strong admixture partially explains the observed heterogeneous landscape of differentiation and suggests that genetic divergence most likely built up in allopatry. In general, genomic outlier windows or islands have been suggested to form more rapidly under secondary contact and erosion of genetic differentiation than under constant gene flow [27,110]. Compared to most benthivorous brown trout populations, individuals from the MCF population show increased admixture and higher genetic ferox ancestry (Figure 2). Despite this, the MCF population is morphologically benthivorous, indicating that ferox trout do not form a genetically clearly distinct group on the genome-wide scale but that outlier regions distinguish ferox trout and benthivorous brown trout.

Such genomic outlier regions (genomic islands) can form in several ways, such as selection or variation in recombination rate and background selection, affecting the degree of differentiation and size of islands [14,15,21]. Most of the observed genomic outlier windows show moderate to high levels of genetic differentiation (Fst between 0.32 and 0.68), suggesting a rather complex genetic basis of local adaptation and reproductive isolation with many loci across the genome involved [9]. Some variants in the population-based genome scans by sampling site were fixed (1 and 5 SNPs depending on the comparison), suggesting that at least some parts of the genome resisted gene flow. However, those are not shared across population pairs, suggesting that these loci do not consistently differentiate populations and most likely do not contribute to the evolution of a ferox life history. The fact that we did not observe shared fixed loci could also stem from the relatively low marker density, and it is likely that several strongly differentiated outlier loci are linked to fixed variants and differentiated through hitchhiking. We further found evidence that selection formed some of these genomic outlier windows. First, we found signs of selection in several loci located within and outside genomic outlier windows, and second reduced diversity within one of the life history forms in some genomic outlier windows, suggesting that selection potentially drove and/or maintains the differentiation in these genomic regions. This has been shown in other species, even if other factors, such as differences in background selection were the main driver of heterogeneous differentiation across the genome [111]. Genes under background selection compared to those involved in speciation are, however, associated with locally reduced effective population size rather than reduced gene flow [112]. Despite evidence for selection, most of the heterogeneous genomic landscape most likely formed due to the highly variable recombination landscape in brown trout [76]. We did not find evidence that variation in gene density drives patterns of genetic diversity and differentiation across the genome, suggesting that other factors, such as recombination rate and mutation rate variation [14], more likely drive patterns of genome-wide variation in brown trout. To fully resolve the impact of different factors, such as recombination rate variation, background selection or divergent selection on the formation of the observed heterogeneous genomic landscape, future studies using more high-density SNP datasets ideally in combination with a high-quality brown trout reference genome [72], and a combination of different empirical and simulation-based approaches will be necessary.

Furthermore, genes located within genomic outlier windows and associated with *pcadapt* outlier loci can give further insights into the role these genomic regions might play in phenotypic divergence and reproductive isolation. However, due to the relatively low density of SNP markers in our study, these results are mostly suggestive and should be interpreted as a starting point for further investigations of the functional genetic basis of the ferox life history in brown trout. The presence of genes associated with growth and development (*pdzrn3*), as well as immune response (*pik3R5*; *tlr2; pip4k2c*; *igsf3*) in genomic outlier windows and loci under selection suggests that phenotypes such as body size, growth rate or immunity are potentially under divergent selection. Similar processes have been associated with eco-morphological and life history differences in other polymorphic species [105,113,114]. Selection on immune response genes between different trophic specialists has been observed in many species, as they are adapted to different parasites due to different foraging habitats and diets [115,116,117]. Furthermore, some genomic outlier windows co-localized with Atlantic salmon-derived QTL for body shape and weight, condition factor and timing of sexual maturation (Figure 4A), which are traits that are divergent between piscivorous ferox trout and benthivorous brown trout [51,52], supporting the hypotheses that these traits are under divergent selection. The possible link of body size and reproductive isolation through size-assortative mating in brown trout [106] further supports the potential role of genes within genomic outlier windows in strengthening reproductive isolation. In addition to genes related to life history, immune response and morphology, genes related to the regulation of (global) gene expression were also under selection and located within genomic outlier windows, suggesting that genetically determined differences in gene expression might play an important role in the ecological and phenotypic divergence. However, more detailed analyses using more high-density genomic data and functional studies are necessary to determine the absolute involvement of these genes in the evolution and maintenance of reproductive isolation and phenotypic divergence.

These results not only contribute to our growing understanding of the genetic basis of reproductive isolation and trophic specialization but also provide important information for the conservation management of ferox trout. The IUCN lists ferox trout as a species separate from brown trout, but recognizes that it is data deficient and more research is needed on the genetic distinctiveness and evolutionary history of ferox trout [60]. Our data demonstrate that ferox trout, at least in the Maree catchment, are clearly genetically distinct from the common benthivorous brown trout and supports the need for targeted conservation efforts, as ferox trout clearly hold distinct and evolutionary unique genetic variation.

There are several limitations to this study. First, the relatively low number of ferox trout and the geographically limited sampling makes it difficult to derive generalities for the evolution of the ferox life history. Realistically, due to the extreme low abundance of ferox trout and difficulties of obtaining high-quality samples, low sample sizes will most likely always be an issue with ferox trout studies. The relatively low density of SNPs (4 SNPs/500kb) potentially affects our ability to identify genomic outlier regions. However, this will most likely lead us to miss genomic outliers but not to falsely identify them. In turn, the relatively low SNP density also leads to low confidence in the identification of candidate genes and biological pathways. Therefore, these results should be interpreted with caution. Most of these problems could be resolved in the future with whole-genome re-sequencing approaches and increasing availability of high-quality genomes.

Overall, understanding the factors driving the formation and maintenance of reproductive isolation and local adaptation, particularly in the face of gene flow, will inevitably require more empirical work on a range of species and populations that display different demographic histories. The replicated and recent postglacial divergences of salmonid species in the northern hemisphere are an ideal system for studying these effects under different demographic scenarios, and the recent availability of high-quality genomic resources for many of these species now makes this feasible. However, more detailed information on the recombination landscape across the genome and its variability among species and populations is necessary. Combining empirical studies in natural populations on the effects of gene flow, hybridization, selection and demographic history with novel theoretical advances and studies on the phenotypic effects of genes will allow researchers to disentangle the effects of different genomic features and evolutionary factors on the origins and maintenance of reproductive isolation.

## Figures and Tables

**Table 1:** Population genetic summary statistics by sampling site.

Location	Code	Life History	N. ddRAD	N. mtDNA	Private	SNPs	H_O_	π
Kernsary	KER	ferox	8	8	99	15,698	0.005	0.006
McFarlane	MCF	bn	6	6	48	15,802	0.006	0.006
Slattadale	SLA	bn	3	3	20	15,219	0.005	0.006
Loch na Fideil	LNF	bn	11	11	197	15,644	0.006	0.006
Grudie	GRU	bn	7	7	111	15,488	0.006	0.006
Taggan	TAG	bn	-	3	-	-	-	-
Loch Lomond	STL	sea trout	-	3	-	-	-	-

Table legend: bn: benthivorous brown trout; Number of individuals genotyped using ddRADseq (N.ddRAD) and number of individuals used for mitochondrial ND1 gene amplification (N.mtDNA); Private: Number of private sites in global ddRADseq dataset; SNPs: Total number of SNPs in global ddRADseq dataset; H_O_**:** Observed heterozygosity. Genetic diversity measures were calculated based on all sites (variant and invariant).

**Table 2:** Analysis of molecular variance based on ddRADseq data.

Marker	Source of Variation	Nested in	% var	F-Stat	F-Value	*p*-Value
ddRADseq	Within ind.	-	85.5	Fit	0.145	-
	Among ind.	Pop	4.3	Fis	0.048	<0.001
	Among sites ^1^	Type	7.2	Fsc	0.074	<0.001
	Among types ^2^	-	3.0	Fct	0.030	0.188

Table legend: ^1^ Sampling sites, ^2^ life history forms (ferox trout and benthivorous brown trout).

**Table 3:** Selection of demographic models.

Model	ln(lhoods) ^1^	N. Parameters	AIC ^2^	∆AIC
SCadm	−109,160.64	8	218,337.28	0
IMchange	−109,198.39	7	218,410.79	73.5
SC	−109,221.83	6	218,455.65	118.4
IM	−109,228.29	5	218,466.57	129.3
AM	−109,230.45	6	218,472.90	135.6
SI	−109,679.11	4	219,366.21	1028.9

Table legend: ^1^ ln(likelihood), ^2^ Akaike information criterion.

**Table 4:** Mean parameter estimates and confidence intervals (CI) for the secondary contact with introgression demographic model.

Parameter	Mean	2.5% CI	97.5% CI
Ancestral Ne	12,326	7955	16,250
Ne brown	1146	1110	1173
Ne ferox	393	376	409
adm (F-B) ^1^	0.817	0.806	0.827
adm (B-F) ^2^	0.536	0.418	0.648
Mig ^3^	0.00220	0.00210	0.00232
Tsc ^4^	469	445	498
Tdiv ^5^	9159	8271	10,292

Table legend: ^1^ Admixture proportion from ferox into benthivorous brown trout; ^1,2^ Admixture proportion from benthivorous brown trout into ferox trout; ^3^ symmetric bi-directional migration rates; ^4^ Timing of secondary contact and ^5^ divergence time in generations. Effective population sizes (Ne) are given in number of diploid individuals.

## Supplementary Materials

The following are available online at http://www.mdpi.com/2073-4425/9/6/280/s1, Supplementary Figures S1–S7, Tables S1–S4.
