# Supplementary material for: The Genetic Architecture Underlying the Evolution of a Rare Piscivorous Life History Form in Brown Trout after Secondary Contact and Strong Introgression
Source: Genes (Basel). 2018 May 31;9(6):280. doi: 10.3390/genes9060280 (PMC6026935; doi:10.3390/genes9060280)
Supplement: Supplementary file 1 [file genes-09-00280-s001.pdf]

The genetic architecture underlying the evolution of a rare piscivorous life history form in brown trout after secondary contact and strong introgression.

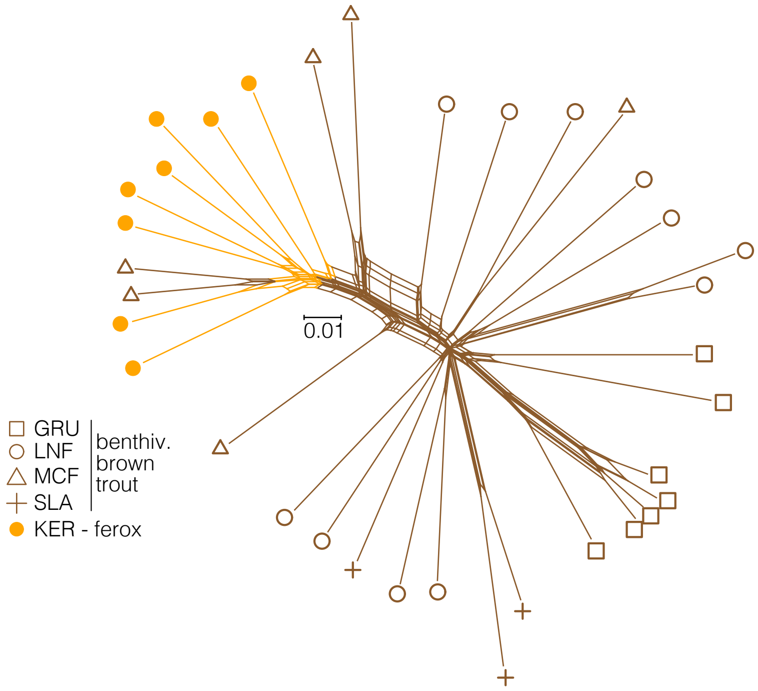

Figure S1. Neighbour-joining network based on 16,066 SNPs.

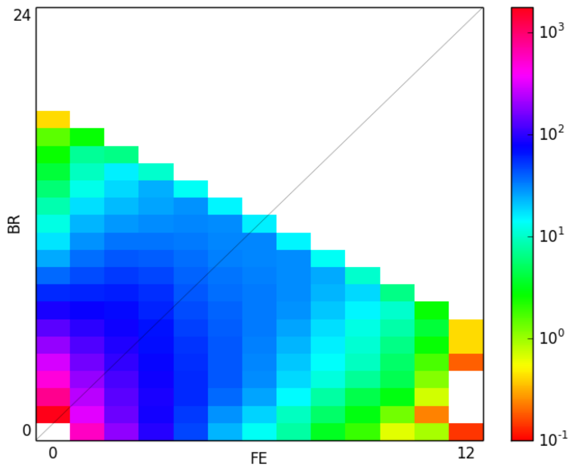

Figure S2. Folded site-frequency spectrum for ferox trout (FE) and combined benthivorous brown trout (BR).

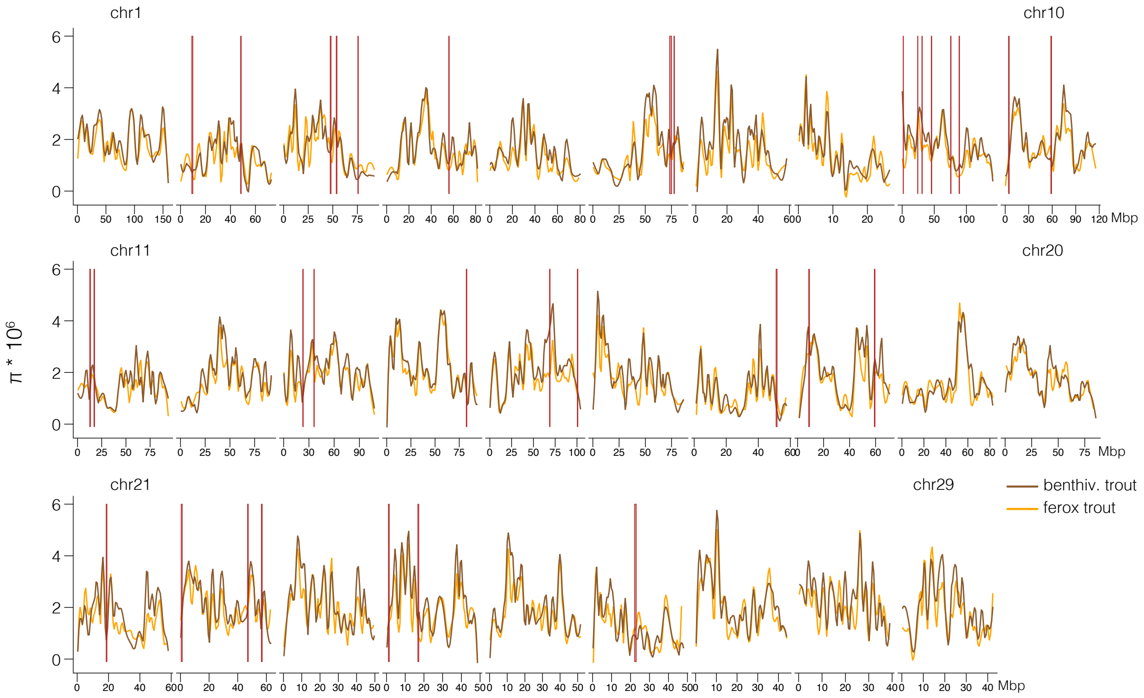

Figure S3. Genome scan of nucleotide diversity (π) across each chromosome for benthivorous brown trout (brown line) and ferox trout (orange line). Genomic islands of divergence are marked with red bars.

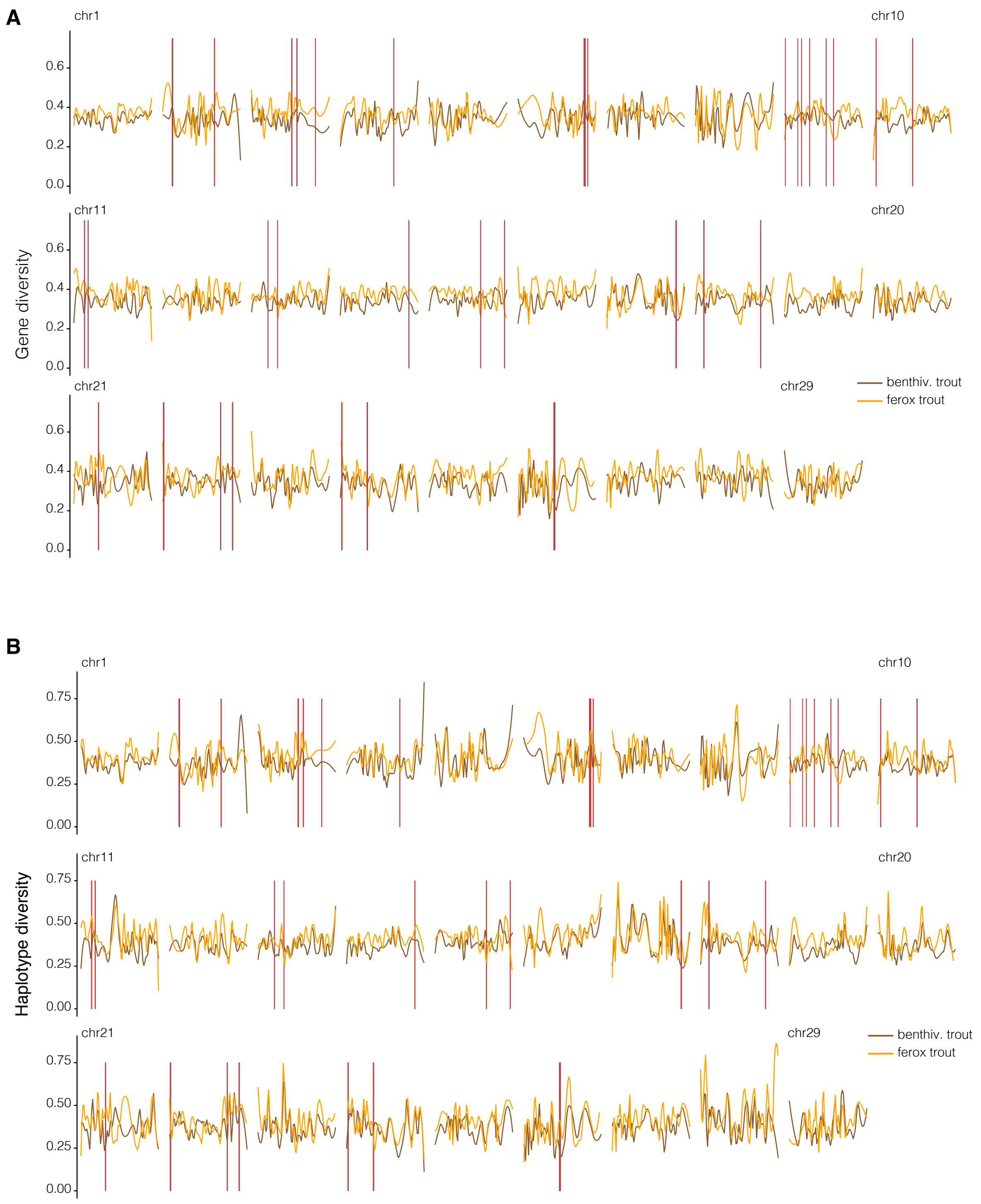

Figure S4. Genome scan of gene diversity and haplotype diversity, both calculated based on haplotype-information derived from full RAD loci. Genomic islands of divergence are marked with red bars.

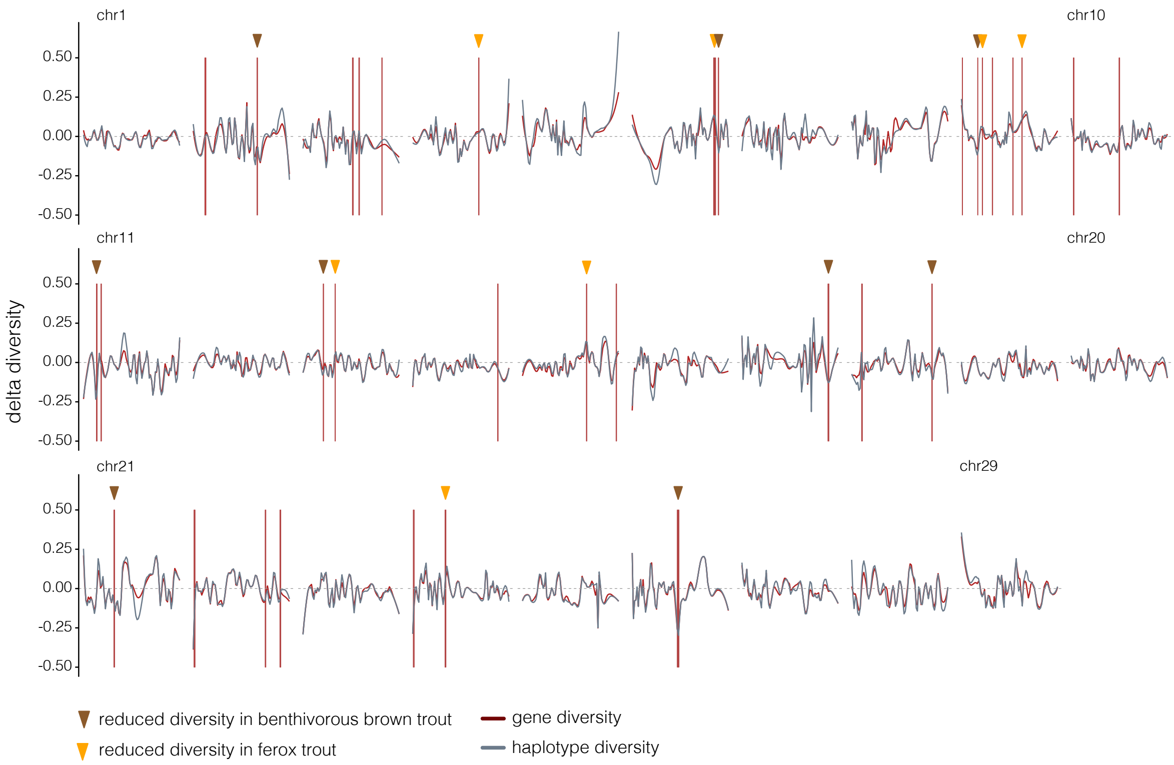

Figure S5. Delta haplotype and gene diversity across the Atlantic salmon genome. Negative delta diversity shows reduced diversity in benthivorous brown trout compared to ferox trout, whereas positive delta diversity shows reduced diversity in ferox trout. Genomic islands are highlighted with red bars. Brown and orange arrow heads highlight genomic islands with reduced diversity in benthivorous brown trout or ferox trout, respectively.

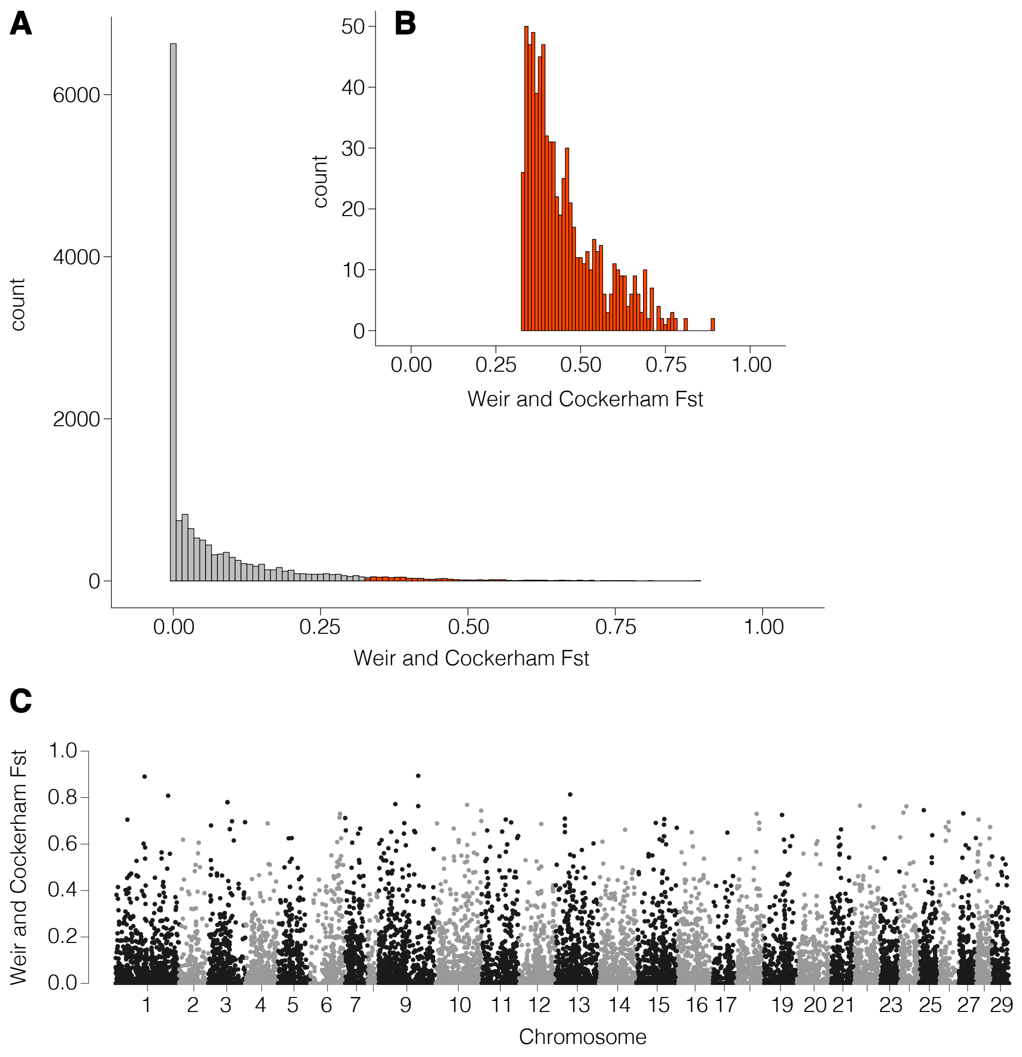

Figure S6. Fst distribution across the genome. **A**) Genome-wide distribution of Fst-values. Top 5% Fst-outlier loci are highlighted in red. **B**) Fst-value distribution of top 5% Fst-outlier loci. **C**) Distribution of Fst-values across the Atlantic salmon genome. Loci are alternatingly coloured by chromosome.

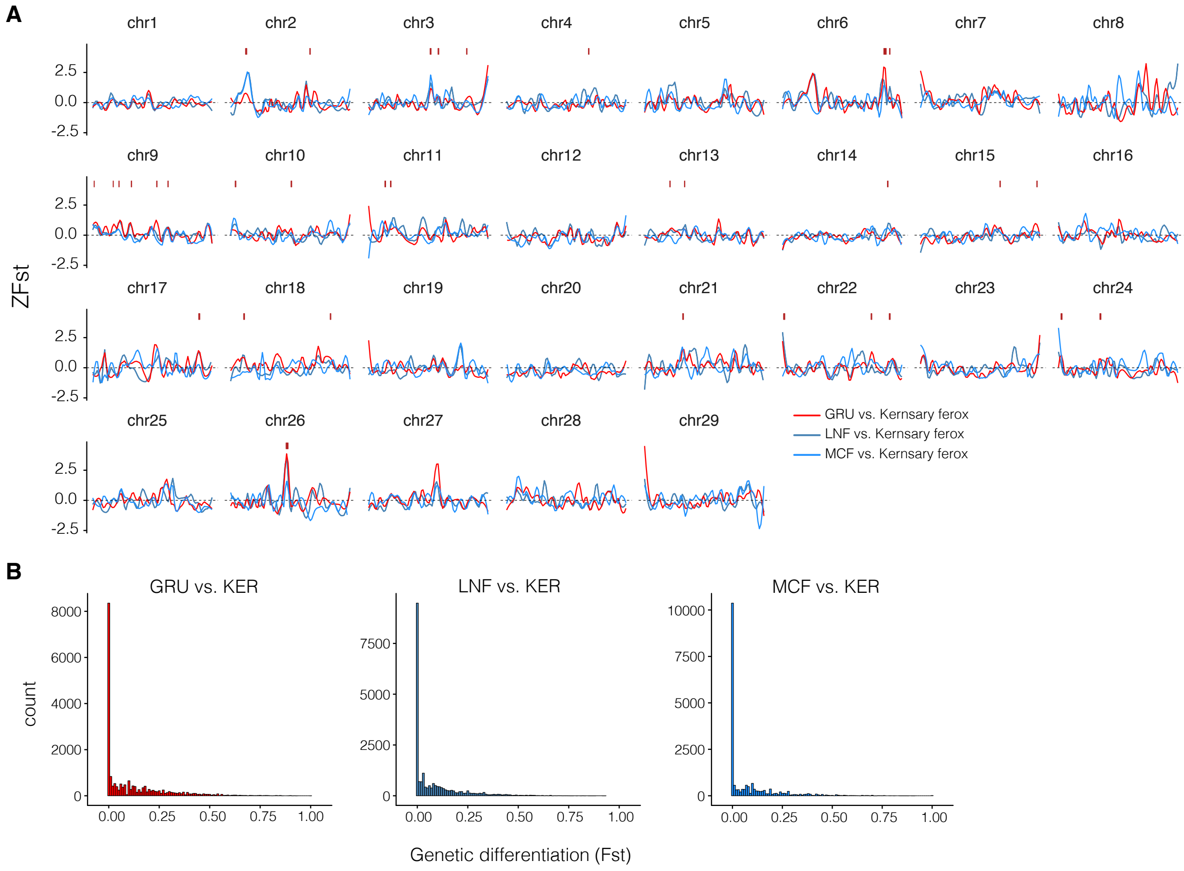

Figure S7. A) Genetic differentiation (loess-smoothed window-based ZFst) across the Atlantic salmon genome for sampling site specific comparisons. Red bars highlight genomic islands identified in the combined genome scan. **B)** Genome-wide distribution of Fst-values (on a SNP basis) by sampling site.

Table S1. Model selection of the most likely demographic model for the reduced dataset.

Model	ln(lhood) [1]	N.parameters	AIC	ΔAIC
**SCadm**	-85481.55	8	170979.1	0.0
**IMchange**	-85486.90	7	170987.8	8.7
**SC**	-85504.60	6	171021.2	42.1
**IM**	-85506.77	5	171023.5	44.4
**AM**	-85506.88	6	171025.8	46.7
**SI**	-85776.11	4	171560.2	581.1

Table legend: [1]ln(likelihood), AIC: Aike information criterion.

Table S2. GO-terms (biological processes) associated with genes within genomic islands. No GO-term was significantly overrepresented (FDR < 0.05).

GO-term	Description	Fold enriched	P-value	FDR	Gene names
GO:0055085	transmembrane transport	2.85	0.0013	1	atp6v1d;mmgt1;slc25a40;slc37a1;slc7a2;slc39a9;chrna5;cyc1;gabrp;chrnb4;grik5
GO:0006401	RNA catabolic process	10.44	0.0029	1	rnaseh1;smg8;cnot7
GO:0034220	ion transmembrane transport	3.09	0.0039	1	mmgt1;slc7a2;slc39a9;chrna5;cyc1;gabrp;chrnb4;grik5
GO:0006810	transport	1.91	0.0048	1	atp6v1d;ergic3;mmgt1;chmp3;slc25a40;slc37a1;slc7a2;slc39a9;ccs;chrna5;ap5s1;cyc1;syt12;gabrp;pom121;chrnb4;grik5
GO:0051234	establishment of localization	1.87	0.0060	1	atp6v1d;ergic3;mmgt1;chmp3;slc25a40;slc37a1;slc7a2;slc39a9;ccs;chrna5;ap5s1;cyc1;syt12;gabrp;pom121;chrnb4;grik5
GO:0006811	ion transport	2.61	0.0068	1	mmgt1;slc7a2;slc39a9;ccs;chrna5;cyc1;gabrp;chrnb4;grik5
GO:0007179	transforming growth factor beta receptor signaling pathway	13.41	0.0096	1	smad5;smad9
GO:0071559	response to transforming growth factor beta	13.41	0.0096	1	smad5;smad9
GO:0071560	cellular response to transforming growth factor beta stimulus	13.41	0.0096	1	smad5;smad9
GO:0010629	negative regulation of gene expression	3.82	0.0097	1	eif4ebp2;smad9;cnot7;brms1la;nrde2

Table S3. KEGG pathways (*Danio rerio*) associated with genes within genomic islands. No KEGG pathway was significantly overrepresented (FDR < 0.05).

KEGG pathway	Description	Fold enriched	P-value	FDR	Gene names
dre04110	Cell cycle	3.406	0.0569	1	e2f1;ccne1;dbf4
dre04068	FoxO signaling pathway	2.749	0.0944	1	cat;pik3r5;agap2
dre04146	Peroxisome	3.561	0.1079	1	cat;crot
dre04080	Neuroactive ligand-receptor interaction	1.929	0.1132	1	lpar1;chrna5;gabrp;chrnb4;grik5
dre00563	Glycosylphosphatidylinositol (GPI)-anchor biosynthesis	6.812	0.1372	1	pigu
dre04350	TGF-beta signaling pathway	3.072	0.1375	1	smad5;smad9
dre00515	Mannose type O-glycan biosynthesis	6.528	0.1427	1	b4gat1
dre04012	ErbB signaling pathway	2.798	0.1596	1	pik3r5;camk2b
dre00514	Other types of O-glycan biosynthesis	5.402	0.1699	1	colgalt1
dre04144	Endocytosis	1.832	0.1716	1	agap1;chmp3;cav2;agap2

Table S4. Genes associated with loci putatively under selection.

Chromosome	Start	End	Annotation AS100	SNP position	Gene name
NC_027300.1	72268063	73360509	macrod2	72742754	MACRO Domain Containing 2
NC_027301.1	48437588	48465607	LOC106587077	48444034	phosphoinositide 3-kinase regulatory subunit 5-like
NC_027306.1	1182340	1211964	LOC106608430	1191948	toll-like receptor 2
NC_027308.1	41684365	41709169	LOC106611358	41703379	potassium channel subfamily K member 2-like
NC_027312.1	35989028	36000630	LOC106567076	35989910	phosphatidylinositol 5-phosphate 4-kinase type-2 gamma
NC_027314.1	69288197	69426575	LOC100380856	69438241	thymocyte selection-associated high mobility group box protein TOX
NC_027316.1	31048572	31051812	LOC106575965	31040284	submaxillary mucin-like protein
NC_027320.1	18801789	18803016	Transmembrane	18800138	Transmembrane protein 50B
NC_027320.1	18894788	18901571	LOC106581898	18865196	ribosomal RNA processing protein 1 homolog B-like
NC_027320.1	21137945	21315616	LOC106581930	21291538	PTB domain-containing engulfment adapter protein 1
NC_027321.1	13654002	13783467	LOC106582849	13766129	E3 ubiquitin-protein ligase PDZRN3-like
NC_027324.1	32744589	32876560	LOC106586624	32836501	immunoglobulin superfamily member 3-like
